# Detection of fever in children emergency care: comparisons of tactile and rectal temperatures in Nigerian children

**DOI:** 10.1186/1756-0500-3-108

**Published:** 2010-04-20

**Authors:** Felix O Akinbami, Adebola E Orimadegun, Olukemi O Tongo, Olubukola O Okafor, Olusegun O Akinyinka

**Affiliations:** 1 Department of Paediatrics, University College Hospital, Ibadan, Nigeria; 2 Institute of Child Health, College of Medicine, University of Ibadan, Ibadan Nigeria

## Abstract

**Background:**

Clinical thermometry is the objective method for temperature measurements but tactile assessment of fever at home is usually the basis for seeking medical attention especially where the cost and level of literacy preclude the use of thermometers. This study was carried out to determine the reliability of tactile perception of fever by caregivers, nurses and house physicians in comparison to rectal thermometry and also the use of commonly practiced surface of the hand in the care of ill children. All caregivers of children aged 6 to 59 months who presented to the emergency department were approached consecutively at the triage stage but 182 children participated. Each child had tactile assessment of fever using palmar and dorsal surfaces of the hand by the caregivers, House Physicians and Nursing Officers. Rectal temperature was also measured and read independently by nurses and house physicians. Comparisons were made between tactile assessments and thermometer readings using a cut-off for fever, 38.0°C and above.

**Findings:**

The caregivers' perception of fever had a sensitivity, specificity, positive predictive value (PPV) and negative predictive value (NPV) of 95%, 23%, 66% and 73%, respectively compared with 93%, 26%, 67% and 69%, respectively for nursing officers. Irrespective of the groups studied, 77.1% of 336 assessors opined that the dorsal surface of the hand was more sensitive in tactile assessment of temperature and the frequently used site for assessment of fever were the head (35.6%) and neck (33.3%). Tactile assessment of temperature over-detected fever in ≥ 24% of cases among the three groups of assessors.

**Conclusions:**

The present study suggests that tactile assessment of temperature may over estimate the prevalence of fever, it does not detect some cases and the need for objective measurement of temperature is emphasised in paediatric emergency care.

## Background

Fever in children is the commonest basis for seeking medical attention in Nigeria, with bacteraemia and malaria parasitaemia reported in 38.2% and 46.1% of febrile infants, respectively [1]. Concerns for fever by parents may be real or imagined ('fever phobia') and therefore assessment and monitoring of temperature is essential for decision making at home and hospital settings [2]. Determination of fever may be subjective and/or objective with caregivers, especially in developing countries relying mainly on tactile perception because of the relatively low level of literacy and the economic cost of reliable thermometers [3]. However, tactile assessment has the tendency to overestimate or underestimate the true core temperature [4]. In a recent study among 126 mothers-child pairs at a paediatric outpatient clinic in Nigeria, 79 out of 82 children who were truly febrile were correctly identified while 25 out of 44 who were non-febrile were reported to have had fever by mothers giving a sensitivity and specificity of 96.3% and 43.2%, respectively [5].

Traditionally, rectal thermometry is the standard for assessment of core temperature and has been shown to correlate well with oesophageal and tympanic thermometry in children [6]. An accurate determination of the absence of fever reassures both parents and health care providers who seek to diminish the fears of fever and therefore inappropriate medical consultation and investigations. The present study was prompted by the fact that a significant proportion of mothers and other caregivers, especially in developing countries rely on tactile evaluations of fever using their hands as means of detection. The present study was therefore undertaken to determine the reliability or otherwise of perception of fever in children as observed by caregivers, physicians and nursing officers and its correlation with rectal temperature. It was also hypothesized that there would be no difference in the sensitivity of palmer and dorsal surfaces of the right hand when used to check for fever and that the health workers will have no preference for any anatomical surface for fever detection.

## Methods

### Study design and sampling

One hundred and eighty-two consecutive patients (whose caregivers gave consent), aged 6 to 59 months, out of total of 203 admissions who presented at the Children Emergency Ward of the University College Hospital Ibadan Nigeria between March and May 2007 were enrolled into the study.

### Data collection procedures

Fever was assessed in all patients by tactile assessment using palmar and dorsal surfaces of the hand by the attending house physicians, nursing officers as well as the caregivers at the triage. Each assessor recorded their findings independently in separate sheets. All assessors placed their hand on the patients for 4 to 6 seconds. Independent recording and observation of tactile assessment of fever (present or not present) as well as the site used by each of the caregivers, the physicians and the Nursing officers was latter collated by one of the investigators in a single study form for each patient.

Similarly, rectal temperatures were taken with the rectal mercury-in-glass thermometer *in-situ *for approximately four minutes and taken out for reading. Measurements were taken with the child placed on his or her stomach to limit struggling. The buttocks were spread out and the thermometer gently inserted about 2.5 cm into the rectum after lubricating the tip of the thermometer. The buttocks were held together to keep the thermometer from falling out. The same thermometer was given to the attending health care providers (house physician and nurse) and independently read and independently recorded thereby minimizing bias. A subset of 112 caregivers, 112 physicians and 112 nursing officers evaluated which side of the hand was better in the tactile assessment of fever and recorded their findings as fever present or absent.

### Data handling

The data collected for each study patient were recorded on a study form with only study numbers. Data was analysed using SPSS 11.0 for Windows (SPSS Inc., Chicago, USA) to calculate sensitivity, specificity, positive predictive values (PPV), negative predictive values (NPV) and degree of agreement using Kappa statistics. Comparisons were made between tactile assessments and thermometer readings using a temperature cut-off for fever at ≥38.0°C. Cross-tabulations were made with reference to determine the relationship between tactile assessment and rectal temperature readings (<*38.0*°C versus ≥*38.0*°C) in the three groups. Categorical variables were compared with Chi-squares while paired *t*-test was used to compare continuous variables. P-values were considered significant for all the hypotheses tested if <0.05. The agreement of tactile assessment was assessed using Kappa statistics according to the scale suggested by Altman [7]. Altman proposed Kappa of less than 0.20 as poor agreement, 0.20 to 0.40 as fair agreement, 0.40 to 0.60 as moderate agreement, 0.60 to 0.80 as good agreement and 0.80 to 1.00 as very good agreement [7].

## Results

The median age of all the patients assessed was 15.0 months (range = 6.0 - 59.0). There was significant correlation (r = 0.783, p = 0.00) in the temperatures recorded by health care providers with a mean (SD) of 38.3(0.8)°C by house physicians and 38.2(0.9)°C by Nursing officers (paired *t *= 0.320, p = 0.749). The overall prevalence of fever (rectal temperature ≥38°C) was 61.5%. Fever detected by touch was reported in 160 (87.9%) patients by caregivers, 157 (86.3%) patients by nurses and 156 (85.7%) patients by house physicians.

Table 1 shows that caregivers' and house physicians jointly reported fever by touch in 149 (81.9%) patients but differed in 18 (9.9%) patients (Kappa = 0.568; 95% CI = 0.383 - 0.711). Caregivers' and Nurses both reported fever by touch in 151 (83.0%) but their assessments differed in 15 (8.2%) of all cases (Kappa = 0.634; 95% CI = 0.449 - 0.766).

**Table 1:** Agreement between Tactile Temperature Assessment of Caregivers vs Doctors and Caregivers vs Nurses

	*Caregivers*								
				
	*Fever*		*No fever*		*All subjects*		*Kappa statistics*		
	
	*n*	*% of all subjects*	*n*	*% of all subjects*	*n*	*% of all subjects*	*Value*	*95% CI*	*P*
Doctors									
Fever	149	81.9	7	3.8	156	85.7	0.568	0.383 - 0.711	0.00
No fever	11	6.0	15	8.2	26	14.3			
Nurses									
Fever	151	83.0	6	3.3	157	86.3	0.634	0.449 - 766	0.00
No fever	9	4.9	16	8.8	25	13.7			

K - Kappa statistics, CI - Confidence Interval

Table 2 compares tactile perception with thermometry and fever (rectal temperature ≥38°C) was correctly detected by physicians in 92.9% (104/112), by nurses in 93.8% (105/112) each and by caregivers in 94.6% (106/112) of patients. Physicians' perception of fever had a sensitivity of 93.0%, specificity of 24.0%, PPV of 66.0% and NPV of 68.0%. Nurses' perception of fever has sensitivity of 94.0%, specificity of 26.0%, PPV of 67.0% and NPV of 72.0%. Caregivers' tactile assessment of fever has sensitivity of 95.0%, specificity of 23.0%, PPV of 66.0% and NPV of 73.0%. The agreement between tactile perceptions of fever by physicians and rectal temperatures was poor (Kappa = 0.195; 95% CI = 0.075 - 0.289). There was fair agreement between the assessments of temperatures by thermometry and reported by caregivers (Kappa = 0.221; 95% CI = 0.102 - 0.309) and nurses (Kappa = 0.201; 95% CI = 0.087 - 0.282).

**Table 2:** Comparisons of Caregivers, Doctors and Nurses Tactile assessment and Rectal Temperature using thermometric method in 182 patients

Tactile Temp	Rectal Temperature °C				*K *(95% CI)	Sensitivity %(95% CI)	Specificity %(95% CI)	PPV %(95% CI)	NPV %(95% CI)
						
	*≥ 38.0*		*<38.0*						
						
	*n*	*%*	*n*	*%*					
Physicians									
Fever	104	92.9	53	75.7	0.195(0.075 - 0.289)	93(89 - 96)	24(18 - 29)	66(63 - 69)	68(68 - 82)
No fever	8	7.1	17	24.3					
Nurses									
Fever	105	93.8	52	74.3	0.221(0.102 - 0.309)	94(90 - 97)	26(19 - 31)	67(64 - 69)	72(54 - 85)
No Fever	7	6.2	18	25.7					
Caregivers									
Fever	106	94.6	54	77.1	0.201(0.087 - 0.282)	95(91 - 97)	23(17 - 27)	66(64 - 68)	73(53 - 87)
No fever	6	5.4	16	12.1					

K - Kappa statistics, CI - Confidence Interval, PPV - positive predictive value, NPV - negative predictive value

The opinions of caregivers, house physicians and nursing officers on which side of the hand was more appropriate for tactile assessment of temperature were as shown in Table 3. Overall, 259 (77.1%) out of 336 assessors opined that the dorsal surface of the hand was more appropriate in detecting fever irrespective of the part of the body used. Although majority (more than 70%) of the assessors considered dorsal surface as the more sensitive side of the hand for detection of fever, there was no significant difference in the sensitivity of palmer and dorsal surfaces of hand reported by the house physicians, nursing officers, and caregivers.

**Table 3:** Opinion of Caregivers, House Physicians and Nursing Officers on the side of the hand more sensitive for tactile assessment of temperature

*Assessors*	*More sensitive side of the hand*			
	
	*Palmar surface*		*Dorsal Surface*	
	
	*n*	*%*	*n*	*%*
House physicians (n = 112)	24	21.4	88	78.6
Nursing officers (n = 112)	31	27.7	81	72.3
Caregivers (n = 112)	22	19.6	90	80.4

**All assessors **(n = 336)	77	22.9	259	87.1

*X*^2 ^= 2.26, df = 2, p = 0.323

The various anatomical parts of patients' body used for temperature assessment by the house physicians, nursing officers, and caregivers were as shown in Table 4. The most frequently used part of the patients' body for detection of fever by touch was the head in 120 (35.6%), the neck in 112(33.3%), the chest in 35 (10.3%) and the abdominal area in 69 (20.7%). The house physicians, nursing officers, and caregivers have significant different opinions on the site of patients' body used for detecting fever. For example most house physicians (46/112 = 41.1%) believe that neck should be used, while most caregivers (50/112 = 44.6%) believe that head should be used.

**Table 4:** Part of patients' body used for temperature assessment

*Assessors*	*Site of patients' body used*							
	
	*Head*		*Neck*		*Chest*		*Abdomen*	
	
	*n*	*%*	*n*	*%*	*n*	*%*	*n*	*%*
House physicians	31	27.6	46	41.1	16	14.3	19	17.0
Nursing officers	39	34.8	27	24.1	19	17.0	27	24.1
Caregivers	50	44.6	39	34.8	0	0.0	23	20.6
All assessors	120	35.7	112	33.3	35	10.4	69	20.6

*X*^2 ^= 28.77, df = 6, p = 0.00

## Discussion

Fever in children detected by caregivers and health care providers may indicate the need for further clinical and laboratory evaluation as it may be associated with potentially serious parasitic, viral and bacterial infections. The 10% rate of decline by mothers to participate in this study was probably a result of the mothers' emotional burden of illness and the need for urgent and decisive care rather than what the mothers considered as time wasting experiment. This could not have altered the findings from the data significantly. The results of this study showed that approximately three out of five children seen at the children emergency unit during the period truly had fever using rectal thermometric method while at least four out of five would be reported to have fever using tactile assessment by physicians, nurses and caregivers. This study also evaluated the agreements between tactile assessment which is the basis for caregivers seeking hospital care and objective rectal thermometry; and demonstrated that tactile assessment of temperature by caregivers and nursing officers could fairly agreed with rectal temperatures. The inter-method reliability of the tactile assessments between the caregivers and either of house physicians and nurses was adjudged as good using the scale proposed by Altman [7].

Though the difference was not statistically significant, caregivers were marginally more likely to correctly detect fever by tactile perceptions than either physicians or nurses. Our data also showed that caregivers reported fever in 94.6% and missed fever in 5.4% of those who had rectal temperature of ≥38.0°C. However, the positive and negative predictive values revealed that caregivers correctly detected large number of children who had fever but at the same time likely to report fever in a number of children who did not have fever. These findings differ from two previous reports by Whybrew et al.,[8] and Einterz and Bates[9] which showed high sensitivity but low positive predictive values. The tactile assessment of caregivers' sensitivity (95%) in the present study is similar to the value reported in a study carried out in Northern Nigeria [5] in which mothers correctly identified 79 (sensitivity of 96.3%) out of 126 children who were truly febrile. However, the predictive value of a positive test (that is mothers detecting fever by palpation when children were truly febrile) of 76.0%, and the predictive value of a negative test (that is mothers did not detect fever by palpation when children were not truly febrile) of 86.4% in the same study for caregivers were remarkably higher than 66% and 73%, respectively obtained in the present study [5]. The differences in methodology make comparisons with our results difficult. Wammanda and Onazi defined fever as axilliary temperatures of 37.2 degree Celsius or more while rectal thermometry was recorded in the present study after four minutes of insertion. However, we are unaware of any previous study that evaluated the appropriate duration of touch for assessing fever. In the present study all assessors placed their hand on the patients for 4 to 6 seconds. Tactile perception is based on discrete cold-sensitive and heat-sensitive spots in the skin with afferents through Aδ and C fibers [10].

The present study supports the utility value of tactile assessment of temperature as in other study,[11] therefore history of fever reported by caregivers should be considered significant for further evaluation. We are unaware of studies comparing tactile assessment of temperature among physicians, nurses and caregivers. The present data showed that the sensitivity of tactile assessment of fever by caregivers was higher than by physicians and nurses but they have the lowest specificity among the three groups. It is not unlikely that these caregivers over reported fever because of anxiety and this may imply an attempt to seek urgent attentions from the health care providers, though this could have been avoided if they could afford one form of thermometry or the other. It is also plausible that the regular contact of caregiver with child has helped in establishing sooner the deviation from the regular normal perception of body temperature. Controversies over the reliability of tactile assessment of temperature have been variously reported; while a study reported that caregivers assessment of fever could be relied upon by health workers,[12] others cautioned about the interpretations and reliability of caregivers' reports [3].

Although the reasons for the use of different sites of the body for tactile assessment of temperature were not explored in this study, it was observed that assessors would readily choose the exposed part of the body. Singhi and Sood [12] reported that palpation of more than one anatomical site had a sensitivity of 100% and specificity of 92%. The present study did not make comparisons between the use of one and more parts of the body. In a previous study, the head and neck were the commonest anatomical sites used for tactile assessment temperature [13]. Though it is known that receptors for detection of heat and temperature are more concentrated on the palmar surface and finger tips,[10] the dorsal surface of the hand was adjudged to be more appropriate by most of our assessors than the palmar surface for tactile assessment of temperature. It was not sufficiently apparent from our data why the assessors chose the dorsal surface of the hand for perceiving fever; a possible explanation may be the apparent increased thickness of the epidermis of the palm compared with the dorsum of the hand which could have led to the believe that it would not be as sensitive as the dorsal surface of the hand.

## Conclusions

The present study suggests that tactile assessment of temperature may significantly over estimate the prevalence of fever, but rarely miss fever, and children who do not feel hot by tactile perception may still have fever. Therefore, it is advisable that doctors and nurses should not disregard or seek to diminish the caregivers' perception of fever. Further, the most frequently used part of the body for tactile assessment of body temperature was the head and neck.

## Competing interests

The authors declare that they have no competing interests.

## Authors' contributions

OOK conceived of the study, led its design and drafting of the manuscript, FOA, OOT and OOO participated in the design of the study, data collection and edited the manuscript. AEO participated in the design of the study, data collection, analyzed the data and edited the manuscript. All authors read and approved the final manuscript.
